# A Case of Pancreatic Cancer with High Tumor Mutation Burden Showing a Remarkable Response to Pembrolizumab

**DOI:** 10.70352/scrj.cr.26-0273

**Published:** 2026-07-03

**Authors:** Kazuto Takata, Yuki Kitano, Rumi Itoyama, Shigeki Nakagawa, Hideaki Miyamoto, Hirohisa Okabe, Hiromitsu Hayashi, Yasuhito Tanaka, Masaaki Iwatsuki

**Affiliations:** 1 Department of Gastroenterological Surgery, Graduate School of Life Sciences, Kumamoto University, Kumamoto, Kumamoto, Japan; 2 Department of Gastroenterology and Hepatology, Graduate School of Life Sciences, Kumamoto University, Kumamoto, Kumamoto, Japan

**Keywords:** pancreatic cancer, microsatellite instability–high, tumor mutation burden–high, immune checkpoint inhibitors, comprehensive genomic profiling

## Abstract

**INTRODUCTION:**

Pancreatic cancer (PC) is an aggressive malignancy with limited therapeutic options and generally poor responsiveness to immune checkpoint inhibitors (ICIs). Microsatellite instability–high (MSI-High) and tumor mutation burden–high (TMB-High) phenotypes are rare, each occurring in approximately 1% of PC cases, but may predict improved response to ICIs. We report a case of recurrent PC harboring both MSI-High and markedly elevated TMB, in which pembrolizumab achieved an exceptional and durable response.

**CASE PRESENTATION:**

A 72-year-old man presented with appetite loss, and contrast-enhanced CT revealed a large pancreatic tail tumor invading the stomach, colon, spleen, and left kidney. Fine-needle biopsy confirmed adenocarcinoma. Despite initial modified FOLFIRINOX therapy showing progressive disease, subsequent gemcitabine plus nab-paclitaxel achieved partial response (PR). However, gastrointestinal bleeding due to gastric invasion necessitated surgery. The patient underwent extensive resection, including distal pancreatectomy with splenectomy, total gastrectomy, partial colectomy, nephrectomy, adrenalectomy, and portal vein resection and reconstruction using the left renal vein. Pathology demonstrated well-differentiated ductal adenocarcinoma with multiorgan invasion and portal vein tumor thrombus (ypT3N1M0, Stage IIB, Evans Grade IIb). Although adjuvant chemotherapy was considered, it could not be initiated because of persistent postoperative diarrhea and delayed recovery of the patient’s general condition. Four months after surgery, recurrence was detected in the para-aortic lymph nodes, with suspected peritoneal dissemination causing right hydronephrosis, accompanied by elevated carbohydrate antigen 19-9 (CA19-9). Comprehensive genomic profiling using the surgically resected tumor specimen revealed MSI-High status and extremely high TMB (75 mutations/Mb), and genomic alterations in *MSH2*, *MSH3*, *KRAS*, *TP53*, *ARID1A*, *ATR*, and *SMAD2*. Pembrolizumab was initiated, leading to rapid normalization of CA19-9 and continuous tumor shrinkage. The best overall response was classified as PR, and this PR has been maintained. At 26 months after surgery, the patient remains on pembrolizumab with sustained clinical benefit and no significant immune-related adverse events.

**CONCLUSIONS:**

This case highlights the potential for exceptional and durable response to pembrolizumab in PC characterized by concurrent MSI-High and markedly elevated TMB. Given the rarity of these biomarkers and the typically poor prognosis of PCs, early genomic profiling may facilitate personalized treatment strategies and improve outcomes in selected patients.

## Abbreviations

CA19-9
: carbohydrate antigen 19-9
CGP
: comprehensive genomic profiling
dMMR
: deficient mismatch repair
ICIs
: immune checkpoint inhibitors
MSI-High
: microsatellite instability–high
ORR
: objective response rate
OS
: overall survival
PC
: pancreatic cancer
PFS
: progression-free survival
PR
: partial response
TMB-High
: tumor mutation burden–high

## INTRODUCTION

PC is one of the most malignant tumors with a poor prognosis. Surgery, chemotherapy, and radiation therapy are used to treat PC, the operable cases count for 10%–20% of all cases, and the 5-year survival rate for all cases being 11.5%.^1)^ In recent years, it has been reported that ICIs are useful in cases of solid cancer with MSI-High. However, MSI-High status is observed in only approximately 1% of PC cases, and although the reported efficacy of ICIs varies among studies, selected patients may achieve durable clinical benefits. Furthermore, TMB-High has been confirmed in approximately 1.1% of PC cases.^2)^ In this report, we report a case of recurrent PC exhibiting both MSI-High and TMB-High, in which pembrolizumab achieved a durable response following curative surgical resection. We also reviewed previous literature to discuss the management and treatment of PC with MSI-High or TMB-High.

## CASE PRESENTATION

A 72-year-old man presented with appetite loss. He had a family history of malignancy, including colorectal cancer in his father and younger sister and gastric cancer in his mother. Contrast-enhanced CT revealed a huge pancreatic tumor occupying the left subphrenic space. The tumor was suspected to invade adjacent organs, including the stomach, transverse colon, and left kidney. The tumor was in contact with approximately one-quarter of the circumference of the portal vein; however, no definite portal vein tumor thrombus was identified on preoperative imaging (**Fig. 1A**). No distant metastases were detected. Fine needle biopsy revealed adenocarcinoma, and chemotherapy was planned. However, the tumor had invaded the transverse colon, resulting in a bowel obstruction. In advance, colostomy with double orifices was performed in the transverse colon, and the patient received chemotherapy with modified FOLFIRINOX for 3 months. Treatment response was progressive disease according to RECIST version 1.1,^3)^ so the patient was switched to gemcitabine plus nab-paclitaxel therapy. The response was PR; however, severe anemia due to the bleeding from gastric invasion developed. The patient was determined to undergo surgery to control the bleeding. The patient underwent distal pancreatectomy with splenectomy, total gastrectomy, partial colectomy, resection of left kidney, partial resection of small intestine, excision of portal vein tumor thrombus, resection and reconstruction of portal vein with left renal vein graft, and regional lymph node dissection (**Fig. 1B**). Pathological findings revealed that 95 × 79 mm of well-differentiated ductal adenocarcinoma invaded the stomach, colon, spleen, left kidney, left adrenal gland, and portal vein, with tumor thrombus formation in the portal vein. Regional lymph node metastases were identified in 3 of 14 dissected nodes. All surgical margins were negative, indicating R0 resection. The pathological stage was ypT3N1M0, ypStage IIB, and the histological response to preoperative therapy was classified as Evans Grade IIb.^4,5)^ The patient was discharged 20 days after the operation, without serious complications. The serum CA19-9 level was 86.8 U/mL at initial presentation. During treatment with modified FOLFIRINOX, the CA19-9 level increased to 179.9 U/mL, consistent with progressive disease. After switching to gemcitabine plus nab-paclitaxel, the CA19-9 level decreased to 108.0 U/mL immediately before surgery and normalized postoperatively to 15.0 U/mL (**Fig. 2**). Although adjuvant chemotherapy was considered because of the pathological stage IIB disease, it could not be initiated because the patient experienced persistent postoperative diarrhea, which delayed recovery of his general condition and nutritional status. Therefore, the patient was followed up closely after surgery.

**Fig. 1:**
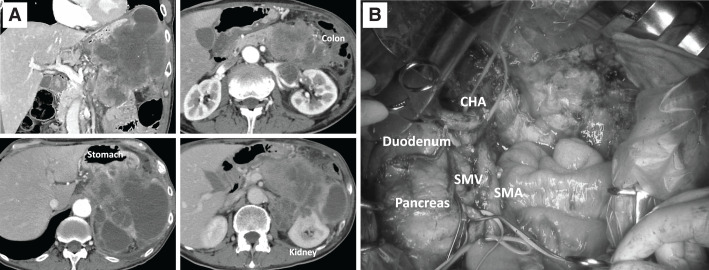
(**A**) Contrast-enhanced CT revealed the hypovascular tumor occupying the left upper abdomen in the pancreatic tail invading the transverse colon, stomach, and left kidney. (**B**) Intraoperative findings after surgical removal of the tumor. CHA, common hepatic artery; SMA, superior mesenteric artery; SMV, superior mesenteric vein

**Fig. 2:**
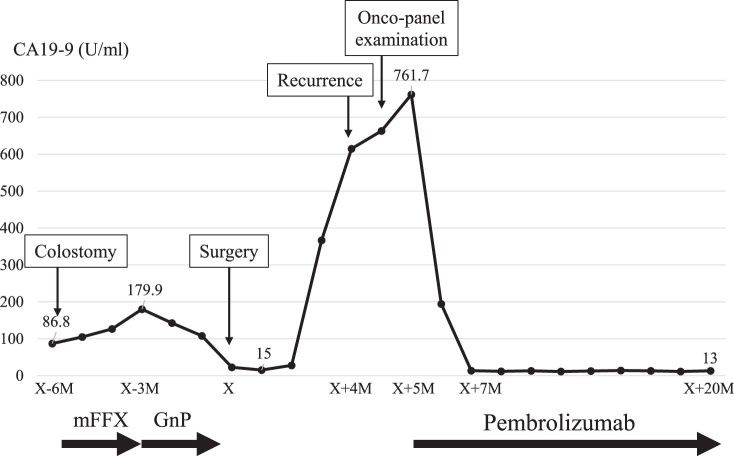
Clinical course and changes in tumor markers. CA19-9, carbohydrate antigen 19-9; GnP, gemcitabine + nab-paclitaxel; mFFX, modified FOLFIRINOX; M, months

Four months after surgery, the serum CA19-9 level increased to 761.7 U/mL, and CT revealed right hydronephrosis and enlarged para-aortic lymph nodes (**Fig. 3A**). The right hydronephrosis was considered to be caused by ureteral stenosis associated with peritoneal dissemination rather than local recurrence, although no definite measurable mass was identified around the ureter. A ureteral stent was placed for the right hydronephrosis. Based on these findings, recurrent diseases with distant metastasis were diagnosed, and CGP was performed using the surgically resected tumor specimen. The analysis revealed MSI-High status, markedly elevated TMB of 75 mutations (mut)/Mb, and genomic alterations in *MSH2*, *MSH3*, *KRAS*, *TP53*, *ARID1A*, *ATR*, and *SMAD2*. Based on these molecular findings, pembrolizumab was initiated. After initiation of pembrolizumab, the CA19-9 level normalized within 2 months, and follow-up CT demonstrated shrinkage of the recurrent lesions (**Fig. 3B**), including the para-aortic lymph nodes. The right hydronephrosis also improved, and the ureteral stent could be removed after response to pembrolizumab. The best overall response was classified as PR, and this response has been maintained. At 26 months after surgery, the patient continues to receive pembrolizumab without significant adverse events and remains alive with sustained disease control.

**Fig. 3:**
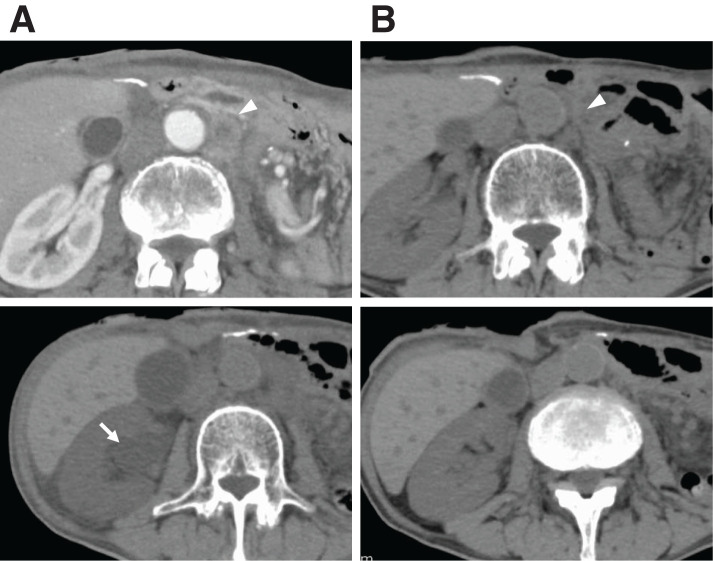
Radiological response of recurrent lesions to pembrolizumab. (**A**) CT at recurrence demonstrated enlarged para-aortic lymph nodes (arrowheads) and right hydronephrosis (arrow) caused by suspected peritoneal dissemination. (**B**) After pembrolizumab treatment, the para-aortic lymph nodes decreased in size and the right hydronephrosis improved.

## DISCUSSION

In recent years, ICIs have demonstrated efficacy in treating solid tumors characterized by MSI-High.^6)^ However, MSI-High status in PC is exceedingly rare, occurring in only 1%–2% of cases, and the reported efficacy of ICIs in MSI-High PC varies among studies. Similarly, TMB-High is also uncommon in PC, identified in approximately 1.1% of cases, with 59.4% of these cases concurrently exhibiting MSI-High status.^7)^ Although even among TMB-High PC, the mean TMB is relatively low at 37.6 mut/Mb,^7)^ the present case notably exhibited a particularly high TMB of 75 mut/Mb.

Clinical trials evaluating ICIs in MSI-High PC have reported variable outcomes. The KEYNOTE-158 trial enrolled 22 patients with PC treated with pembrolizumab, reporting an ORR of 18.2%, median PFS of 2.1 months, and median OS of 4.1 months.^2)^ In contrast, the retrospective European cohort study, which included 31 patients with advanced MSI-High PC, reported an ORR of 48.4% and a median PFS of 26.7 months.^8)^ These results suggest that while ICIs may benefit a subset of MSI-High PC patients, the overall response remains limited. To clarify the clinical context of the present case, we reviewed previously reported cases of PC with specific genomic or mismatch repair–related features, including MSI-High, TMB-High, and dMMR, that showed favorable responses to ICIs.^9–15)^ As summarized in **Table 1**, these reports suggest that PC harboring MSI-High, TMB-High, or dMMR status may represent a small but clinically important subgroup that can derive substantial benefit from ICIs. Several case reports have indicated that ICIs may be particularly effective in PC characterized by concurrent MSI-High and TMB-High status.^8,16,17)^ Biomarker analysis from KEYNOTE-158, excluding PC, demonstrated an ORR of 29.4% (complete response: 3.9%; PR: 25.5%) in TMB-High (≥10 mut/Mb) patients, compared to 6.3% in those with low TMB (<10 mut/Mb).^2)^ Among 21932 PC patients who underwent CGP, the median OS in the TMB-High group was 25.7 months, which was significantly longer than that in the TMB-Low group (5.2 months) (hazard ratio [HR] = 0.32, p = 0.034). Additionally, in the TMB-High group, ICI therapy resulted in a significantly longer OS compared to other treatments (chemotherapy, etc.) (25.7 vs. 6.6 months, HR = 0.31, p = 0.043).^18)^

**Table 1:** Reported cases of PC with MSI-High, TMB-High, or dMMR status showing favorable responses to ICIs

Authors	Age	Sex	Tumor location	Surgery	Tumor status	MSI	TMB (muts/Mb)	dMMR	Other	Treatment	OS (months)	Reference
Cox et al.	56	M	Pb	−	Locally advanced	High	NA	+		Pembrolizumab	12	^9)^
Zhang et al.	56	M	Ph	+	Recurrence	Low	49.92	−		Sintilimab	29	^10)^
Zhao et al.	51	F	Ph	+	Locally advanced	NA	NA	+	*MLH1*, *PMS2*	Pembrolizumab	48	^11)^
Zhu et al.	61	M	Pt	+	Metastasis	Low	11.7	−		Pembrolizumab + nab-paclitaxel	24	^12)^
Anisetti et al.	75	F	Ph	+	Metastasis	High	NA	NA		Pembrolizumab	18	^13)^
Dai et al.	55	M	Pb	−	Metastasis	NA	68.74	NA		Pembrolizumab	21.7	^14)^
Han and Borazanci	57	M	Ph	+	Metastasis	High	28	NA	*BRCA2*	Ipilimumab/nivolumab	36	^15)^
Present case	72	M	Pt	+	Recurrence	High	75	+	*MSH2*, *MSH3*	Pembrolizumab	26	—

*BRCA*, breast cancer susceptibility gene; dMMR, deficient mismatch repair; F, female; ICIs, immune checkpoint inhibitors; M, male; *MLH*, mutL homolog; *MSH*, mutS homolog; MSI-High, microsatellite instability–high; muts, mutations; NA, not available; OS, overall survival; Pb, pancreatic body; PC, pancreatic cancer; Ph, pancreatic head; *PMS*, postmeiotic segregation increased; Pt, pancreatic tail; TMB-High, tumor mutation burden–high

The exceptionally high TMB observed in the present case may be partly explained by the MSI-High phenotype, which reflects impaired mismatch repair and the accumulation of replication errors.^19)^ In addition, CGP identified alterations in *MSH2* and *MSH3*, both of which are involved in mismatch repair, as well as mutations in *KRAS*, *TP53*, *ARID1A*, *ATR*, and *SMAD2*. Among these alterations, *ARID1A* and *ATR* may be particularly relevant to the highly mutated phenotype, because *ARID1A* deficiency has been associated with impaired mismatch repair, MSI, increased TMB, and enhanced tumor immunogenicity,^20)^ whereas ATR is involved in the DNA damage response pathway.^21)^ Therefore, although MSI-High status is likely the major contributor to the markedly elevated TMB in this case, additional alterations affecting DNA repair or genomic stability may also have contributed. The coexistence of MSI-High, markedly elevated TMB, and DNA repair–related alterations may have created a highly immunogenic tumor microenvironment, potentially explaining the exceptional and durable response to pembrolizumab. The patient also had a notable family history of malignancy, including colorectal cancer in his father and younger sister and gastric cancer in his mother. Given the MSI-High phenotype, MMR-related alterations involving *MSH2* and *MSH3*, and this family history, the possibility of a hereditary cancer syndrome, particularly Lynch syndrome, should be considered.^22,23)^ However, because the genomic alterations were identified by tumor-based CGP, and neither germline genetic testing of the patient nor genetic testing of his family members was performed, we could not determine whether these alterations represented germline or somatic events.^24)^

In the present case, pembrolizumab was administered as the first systemic therapy after postoperative recurrence. However, the patient had already received 2 cytotoxic chemotherapy regimens, modified FOLFIRINOX and gemcitabine plus nab-paclitaxel, before surgery. Because gemcitabine plus nab-paclitaxel had achieved PR preoperatively, re-challenge with this regimen could have been a reasonable treatment option at recurrence. Nevertheless, CGP at recurrence revealed MSI-High status and a markedly elevated TMB of 75 mut/Mb, both of which suggested potential sensitivity to ICI blockade. In addition, adjuvant chemotherapy could not be initiated because of persistent postoperative diarrhea and delayed recovery of the patient’s general condition. Therefore, pembrolizumab was selected as a biomarker-driven treatment rather than re-administration of cytotoxic chemotherapy. This treatment decision reflects the potential clinical importance of early genomic profiling in selecting personalized therapy for recurrent PC.

Currently, the optimal timing for CGP remains undefined. In Japan, CGP is typically reserved for patients who have exhausted standard treatment options or have rare cancers without established standard treatments.^25)^ Consequently, in this case, CGP was performed only after 2 prior chemotherapy regimens. However, emerging evidence suggests that earlier implementation of CGP and subsequent targeted immunotherapy may yield improved clinical outcomes.^8,25,26)^ Given the highly aggressive nature of PC and its poor prognosis, earlier genomic profiling and precision medicine could significantly alter the clinical course, enabling individualized treatment and potentially enhancing survival. In the present case, the treatment course may have been different if CGP had been performed before an initial treatment. This case highlights the need to reconsider the timing of CGP in PCs. Early implementation may identify actionable mutations sooner, enabling personalized treatment and potentially improving outcomes.

## CONCLUSIONS

This case highlights the potential for exceptional and durable response to pembrolizumab in PC characterized by concurrent MSI-High and markedly elevated TMB. The coexistence of MSI-High status, markedly elevated TMB, and MMR-related or DNA repair–associated genomic alterations may define a rare but clinically important subgroup of PC that can derive substantial benefit from ICI blockade. Given the rarity of these biomarkers and the typically poor prognosis of PC, early genomic profiling may facilitate personalized treatment strategies and improve outcomes in selected patients.

## DECLARATIONS

### Use of artificial intelligence tools

No artificial intelligence (AI) tools, including large language models or machine learning software, were used in the preparation, analysis, or presentation of this manuscript.

### Availability of data and materials

The data that support the findings of this study are available from the corresponding author upon reasonable request.

## References

[1] Del Chiaro M, Sugawara T, Karam SD, et al. Advances in the management of pancreatic cancer. BMJ 2023; 383: e073995. 38164628 10.1136/bmj-2022-073995

[2] Marabelle A, Le DT, Ascierto PA, et al. Efficacy of pembrolizumab in patients with noncolorectal high microsatellite instability/mismatch repair-deficient cancer: results from the phase II KEYNOTE-158 study. J Clin Oncol 2020; 38: 1–10. 31682550 10.1200/JCO.19.02105 PMC8184060

[3] Eisenhauer EA, Therasse P, Bogaerts J, et al. New response evaluation criteria in solid tumours: revised RECIST guideline (version 1.1). Eur J Cancer 2009; 45: 228–47. 19097774 10.1016/j.ejca.2008.10.026

[4] Evans DB, Rich TA, Byrd DR, et al. Preoperative chemoradiation and pancreaticoduodenectomy for adenocarcinoma of the pancreas. Arch Surg 1992; 127: 1335–9. 1359851 10.1001/archsurg.1992.01420110083017

[5] Brierley JDGM, Wittekind C. TNM Classification of Malignant Tumors, 8th ed. Wiley: Blackwell; 2017.

[6] Le DT, Durham JN, Smith KN, et al. Mismatch repair deficiency predicts response of solid tumors to PD-1 blockade. Science 2017; 357: 409–13. 28596308 10.1126/science.aan6733 PMC5576142

[7] Lawlor RT, Mattiolo P, Mafficini A, et al. Tumor mutational burden as a potential biomarker for immunotherapy in pancreatic cancer: systematic review and still-open questions. Cancers (Basel) 2021; 13: 3119. 34206554 10.3390/cancers13133119 PMC8269341

[8] Taïeb J, Sayah L, Heinrich K, et al. Efficacy of immune checkpoint inhibitors in microsatellite unstable/mismatch repair-deficient advanced pancreatic adenocarcinoma: an AGEO European Cohort. Eur J Cancer 2023; 188: 90–7. 37229836 10.1016/j.ejca.2023.04.012

[9] Cox RE Jr., Mahipal A, Chakrabarti S. A patient with locally advanced mismatch-repair-deficient pancreatic ductal adenocarcinoma successfully treated with neoadjuvant immunotherapy. Cureus 2021; 13: e14640. 34055508 10.7759/cureus.14640 PMC8144075

[10] Zhang F, Li X, Liu H, et al. Unexpected favorable outcome to sintilimab monotherapy in a relapse pancreatic ductal adenocarcinoma patient with high tumor mutational burden: a case report. Anticancer Drugs 2022; 33: 975–8. 35946538 10.1097/CAD.0000000000001334 PMC9481288

[11] Zhao L, Singh V, Ricca A, et al. Survival benefit of pembrolizumab for patients with pancreatic adenocarcinoma: a case series. J Med Cases 2022; 13: 240–3. 35655630 10.14740/jmc3918 PMC9119368

[12] Zhu PF, Chen YW, Wang MX, et al. Tumor-mutation burden as a marker for immunotherapy of pancreatic cancer: the case report and literature review. Anticancer Drugs 2022; 33: e822–7. 34459462 10.1097/CAD.0000000000001232 PMC8670360

[13] Anisetti B, Coston TW, Ahmed AK, et al. An excellent response of microsatellite instability-high pancreatic adenocarcinoma to pembrolizumab treatment: the role of circulating tumor DNA testing. Cureus 2023; 15: e37239. 37168199 10.7759/cureus.37239 PMC10166407

[14] Dai M, Sheng J, Zhang Q, et al. Case report: partial response to single-agent pembrolizumab in a chemotherapy-resistant metastatic pancreatic cancer patient with a high tumor mutation burden. Front Oncol 2023; 13: 1118633. 36937383 10.3389/fonc.2023.1118633 PMC10016350

[15] Han MY, Borazanci E. A rare case of sporadic mismatch repair deficient pancreatic ductal adenocarcinoma that responded to ipilimumab and nivolumab combination treatment: case report. J Gastrointest Oncol 2023; 14: 458–62. 36915432 10.21037/jgo-22-587 PMC10007950

[16] Ngo P, Shanshal M, Rojan A. Immunotherapy in pancreatic cancer and the importance of tumour testing. BMJ Case Rep 2020; 13: e235774. 10.1136/bcr-2020-235774 PMC7368477 32675128

[17] Grant RC, Denroche R, Jang GH, et al. Clinical and genomic characterisation of mismatch repair deficient pancreatic adenocarcinoma. Gut 2021; 70: 1894–903. 32933947 10.1136/gutjnl-2020-320730

[18] Quintanilha JCF, Storandt MH, Graf RP, et al. Tumor mutational burden in real-world patients with pancreatic cancer: genomic alterations and predictive value for immune checkpoint inhibitor effectiveness. JCO Precis Oncol 2023; 7: e2300092. 37410975 10.1200/PO.23.00092 PMC10581638

[19] Li K, Luo H, Huang L, et al. Microsatellite instability: a review of what the oncologist should know. Cancer Cell Int 2020; 20: 16. 31956294 10.1186/s12935-019-1091-8 PMC6958913

[20] Shen J, Ju Z, Zhao W, et al. ARID1A deficiency promotes mutability and potentiates therapeutic antitumor immunity unleashed by immune checkpoint blockade. Nat Med 2018; 24: 556–62. 29736026 10.1038/s41591-018-0012-z PMC6076433

[21] Saldivar JC, Cortez D, Cimprich KA. The essential kinase ATR: ensuring faithful duplication of a challenging genome. Nat Rev Mol Cell Biol 2017; 18: 622–36. 28811666 10.1038/nrm.2017.67 PMC5796526

[22] Giardiello FM, Allen JI, Axilbund JE, et al. Guidelines on genetic evaluation and management of Lynch syndrome: a consensus statement by the US Multi-Society Task Force on colorectal cancer. Gastroenterology 2014; 147: 502–26. 25043945 10.1053/j.gastro.2014.04.001

[23] Bonadona V, Bonaïti B, Olschwang S, et al. Cancer risks associated with germline mutations in *MLH1*, *MSH2*, and *MSH6* genes in Lynch syndrome. JAMA 2011; 305: 2304–10. 21642682 10.1001/jama.2011.743

[24] Li MM, Datto M, Duncavage EJ, et al. Standards and guidelines for the interpretation and reporting of sequence variants in cancer: a joint consensus recommendation of the Association for Molecular Pathology, American Society of Clinical Oncology, and College of American Pathologists. J Mol Diagn 2017; 19: 4–23. 27993330 10.1016/j.jmoldx.2016.10.002 PMC5707196

[25] Matsubara J, Mukai K, Kondo T, et al. First-line genomic profiling in previously untreated advanced solid tumors for identification of targeted therapy opportunities. JAMA Netw Open 2023; 6: e2323336. 37459099 10.1001/jamanetworkopen.2023.23336 PMC10352863

[26] Thapa B, Ahmed G, Szabo A, et al. Comprehensive genomic profiling: does timing matter? Front Oncol 2023; 13: 1025367. 36865796 10.3389/fonc.2023.1025367 PMC9971445

